# Prevalence of hyperglycemia among adults with newly diagnosed HIV/AIDS in China

**DOI:** 10.1186/1471-2334-13-79

**Published:** 2013-02-11

**Authors:** Yinzhong Shen, Zhenyan Wang, Li Liu, Renfang Zhang, Yufang Zheng, Hongzhou Lu

**Affiliations:** 1 Department of Infectious Diseases, Shanghai Public Health Clinical Center, Fudan University, Shanghai 201508, China

**Keywords:** Acquired immunodeficiency syndrome, Hyperglycemia, Impaired fasting glucose, Diabetes, CD4^+^T lymphocyte count

## Abstract

**Background:**

The prevalence of hyperglycemia among HIV-infected persons who are not receiving antiretroviral therapy is unknown. We conducted a cross-sectional survey to estimate the prevalence of hyperglycemia among Chinese adults with newly diagnosed HIV/AIDS.

**Methods:**

Two thousand and six newly diagnosed HIV/AIDS patients from 10 provinces and municipalities in China were selected during 2009 to 2010. After an overnight fast, serum samples were collected to measure glucose concentrations. Demographics and medical histories were recorded. Factors associated with the presence of diabetes were analysed by logistic regression.

**Results:**

Among the 2006 patients, 75.67% were male. Median age was 40 years (range: 18–86 years). 19.99% had hyperglycemia, 9.47% had impaired fasting glucose (IFG) and 10.52% had diabetes. The prevalences of hyperglycemia, of IFG and of diabetes were 21.54%, 10.28% and 11.27% among men and 15.16%, 6.97% and 8.20% among women, respectively. The prevalence of diabetes increased with increasing age (7.00%, 13.36% and 21.21% among patients who were 18–40, 40–60, and ≥60 years of age respectively) and with decreasing CD4 count (6.74%, 8.45%, 9.69%, and 12.66% among patients with CD4 count of ≥350, 200–350, 50–200, and < 50/mm^3^ respectively). The prevalence of diabetes was higher among ethnic minority patients than among the Han patients (14.37% versus 9.24%). The logistic analysis showed that older age, lower CD4 count and minority ethnicity were significantly associated with an increased risk of diabetes.

**Conclusions:**

Hyperglycemia is highly prevalent among Chinese adults with newly diagnosed HIV/AIDS. Older age, lower CD4 count and minority ethnicity are associated with increased risk of diabetes. All newly diagnosed HIV/AIDS individuals should be routinely evaluated for hyperglycemia.

## Background

AIDS is one of the major public health problems in China. With the widespread use of antiretroviral therapy (ART) in clinical practice, the prognosis and quality of life of HIV/AIDS patients have been significantly improved, and mortality and morbidity from HIV and its complications have dramatically declined [1]. However, the incidence of some non-AIDS-related diseases such as cardiovascular diseases shows a rising trend, and these diseases have become a major cause of illness in patients with HIV [2-4].

Abnormal glucose metabolism that occurs during the course of HIV infection and its treatment is a prevalent condition [5]. Diabetes is a known complication of ART, being associated with exposure to some antiretroviral drugs [6,7]. The reported prevalence of diabetes in HIV-infected populations ranges from 2% to 14%, with differences in prevalence explained by differences in demographic characteristics, lifestyle, and antiretroviral exposure [7]. With the aging of the HIV-positive population, brought about by an increased life expectancy after the widespread use of ART, the prevalence of diabetes is likely to increase. To date, the reported prevalence of diabetes has only been estimated among the population receiving ART, the prevalence and the associated risk factors for diabetes among newly diagnosed HIV/AIDS patients without ART exposure have not been determined.

Diabetes has become a major public health problem in China. A national survey conducted in 2008, involving 46,239 Chinese adults, 20 years of age or older, from 14 provinces and municipalities, showed that the age-standardized prevalence of total diabetes and of pre-diabetes were 9.7% and 15.5%, respectively [8]. However, so far the prevalence of hyperglycemia among newly diagnosed HIV/AIDS patients in China has not been extensively studied. Accordingly, the purpose of the present study was to estimate the prevalence of hyperglycemia among adults with newly diagnosed HIV/AIDS in China, and to identify demographic and HIV-related factors that were associated with the presence of diabetes.

## Methods

### Study population

We conducted a cross-sectional survey on HIV/AIDS in China’s HIV epidemic provinces and municipalities including Guangxi, Yunnan, Henan, Xinjiang, Jiangxi, Guangdong, Shaanxi, Hunan, Heilongjiang and Shanghai during 2009 to 2010. The survey subjects were newly diagnosed HIVAIDS patients who had not received ART. Subjects aged 18 years or more at the time of enrolment with documented HIV-1 infection were eligible for this study. Only antiretroviral naïve subjects were included in the study. Being on ART was an exclusion criterion. Antiretroviral experienced but currently untreated subjects were not eligible for this study. All patients were confirmed to be positive for HIV antibody through laboratory detection, and the diagnosis was in line with national HIV/AIDS diagnostic criteria.

### Blood samples

After at least 10 hours of overnight fasting, a venous blood specimen was collected in a vacuum tube containing sodium fluoride, for the measurement of plasma glucose. Written informed consent was obtained for all subjects; consent forms and procedures, as well as survey protocol, were approved by the Shanghai Public Health Clinical Center Ethics Committee. Plasma glucose was measured with the use of a hexokinase enzymatic method, at the clinical biochemical laboratories in each province. All the study laboratories successfully completed a standardization and certification program.

### Study-outcome definitions

Results of plasma glucose testing were categorized as follows: hyperglycemia (fasting glucose level ≥ 6.1 mmol/l [110 mg/dl]), impaired fasting glucose (IFG) (fasting glucose level ≥ 6.1 mmol/l [110 mg/dl] and <7.0 mmol/l [126 mg/dl]), and diabetes (fasting glucose level ≥7.0 mmol/l [126 mg/dl]). Hyperglycemia was thus defined as either IFG or diabetes.

### Data collection

Data were collected according to standardised criteria. On enrolment, standardised data collection forms were completed at the sites providing information from patient interview and patient case notes. Data collected on newly identified cases included demographic information, risk-behavior information (injection drug use, history of heterosexual or homosexual sex, receipt of blood transfusion), and laboratory test results. Variables of interest included age, sex, race, ethnicity, HIV transmission route, and CD4 count. Age was denoted as <40, 40–60 or ≥60 years. Race/ethnicity was designated as Han or other (minority groups). HIV transmission route was categorized as sexual contact (including homosexual or heterosexual), blood (including blood transfusion or injection drug use), or unknown transmission risk. CD4 count was denoted as <50, 50–200, 200–350 or ≥350/mm^3^.

### Statistical analysis

SPSS software for Windows (version 11.5) was used for statistical analysis. A chi-square test was applied for categorical attributes. We evaluated if the prevalence of diabetes increased with decreasing CD4 count using a chi-square test for trend. Multivariate logistic regression models were used to analyze the association between diabetes and relevant covariates. All variables included in the models were determined a priori based on epidemiological importance and biological plausibility. Variables included in the models were age, sex, ethnicity, CD4 count, and HIV transmission route. Primary analysis was done based on all persons who met inclusion criteria. Sensitivity analyses were performed for patients who had a baseline CD4 count available. The statistical test was two-tailed and performed at a level of statistical significance of 0.05.

## Results

### Patient characteristics

We included a total of 2006 adults with newly diagnosed HIV/AIDS. Table 1 describes the basic characteristics of the study population. The study sample was primarily male (75.67%), the median age was 40 years (41 years for males, 38 years for females), 24.47% were ethnic minorities, and the median CD4 count was 136 cells/mm^3^. Most patients acquired HIV through sexual contact (73.73%).

**Table 1:** Basic characteristics of 2006 newly diagnosed HIV/AIDS patients in China

**Characteristic**	**Patient no. (%)**
Age, years	
<40	1100 (54.84)
40–60	741 (36.94)
≥60	165 (8.22)
Median age (range)	40 (18, 86)
Sex	
Male	1518 (75.67)
Female	488 (24.33)
HIV transmission category	
Sexual contact	1479 (73.73)
Blood	351 (17.48)
Unknown transmission risk	176 (8.79)
CD4 count*, cells/mm^3^	
<50	806 (40.91)
50-200	619 (31.42)
200–350	367 (18.63)
≥350	178 (9.04)
Median CD4 count (range)	136 (1, 891)
Ethnicity	
Han	1515 (75.53)
Other (minority)	491 (24.47)

* 1970 patients had a baseline CD4 count.

### Prevalence of hyperglycemia among men and women with HIV/AIDS

**Figure 1:**
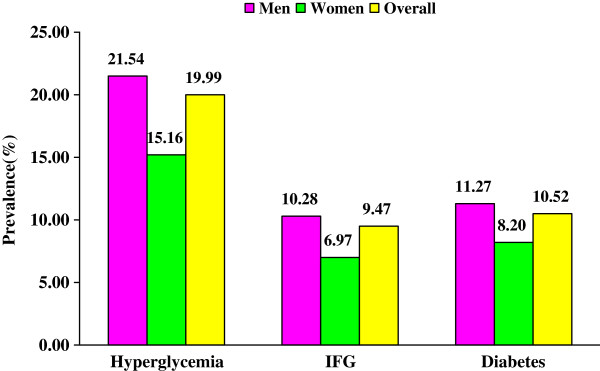
Prevalence of hyperglycemia, IFG and diabetes among men and women with newly diagnosed HIV/AIDS.

Figure 1 describes the prevalence of hyperglycemia, of IFG and of diabetes among men and women with HIV/AIDS. Among the 2006 patients, 401 (19.99%) had hyperglycemia, 190 (9.47%) had IFG, 211 (10.52%) had diabetes. Among 1518 male patients, 327 (21.54%) had hyperglycemia, 156 (10.28%) had IFG, and 171 (11.27%) had diabetes; among 488 female patients, 74 (15.16%), 34 (6.97%) and 40 (8.20%) had hyperglycemia, IFG and diabetes respectively. The prevalence of hyperglycemia and of IFG among men were significantly higher than those among women (*P* = 0.002, * P* = 0.030), the prevalence of diabetes was slightly higher among men than among women (*P* = 0.055).

### Prevalence of hyperglycemia in patients with different CD4 count

**Figure 2:**
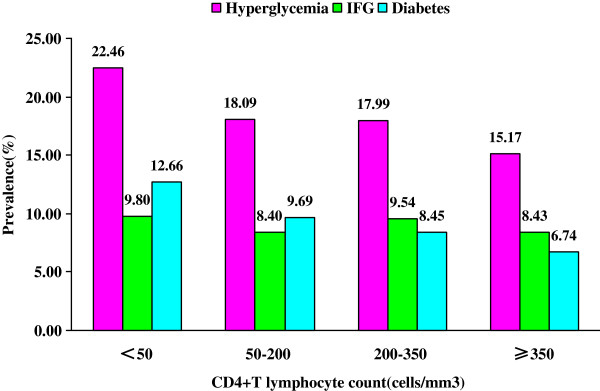
**Prevalence of hyperglycemia, IFG and diabetes among patients with different CD4**^**+**^**T lymphocyte count (1970 patients had a baseline CD4 count).**

Figure 2 describes the prevalence of hyperglycemia, of IFG and of diabetes among patients with different CD4 counts. The prevalence of IFG was 8.43%, 9.54%, 8.40%, and 9.80% among patients with CD4 counts of ≥ 350, 200–350, 50–200, and < 50/mm^3^, respectively. The prevalence of IFG did not differ significantly according to CD4 count (*P* = 0.801). The prevalence of diabetes was 6.74%, 8.45%, 9.69%, and 12.66% among patients with CD4 counts of ≥ 350, 200–350, 50–200, and < 50/mm^3^, respectively. The prevalence of diabetes increased with decreasing CD4 count (*P* = 0.032).

### Prevalence of hyperglycemia among men and women with different age

**Figure 3:**
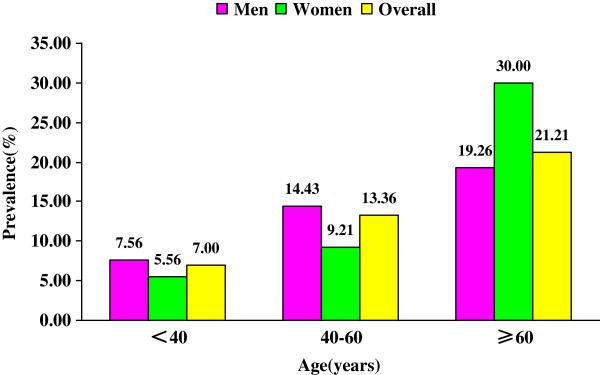
Prevalence of diabetes among men and women according to age.

**Figure 4:**
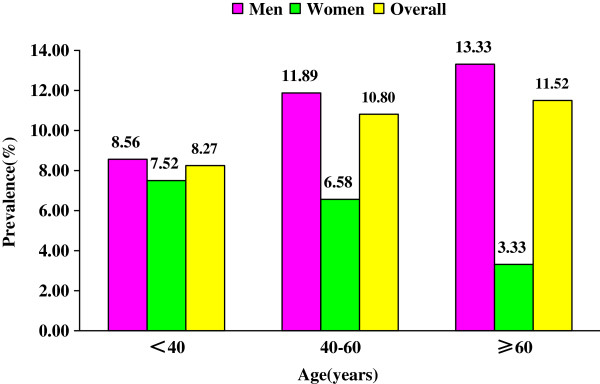
Prevalence of IFG among men and women according to age.

Figure 3 and Figure 4 describe the prevalence of diabetes and of IFG among adults according to age. The prevalence of diabetes was 7.00%, 13.36% and 21.21% among patients who were 18–40, 40–60, and ≥ 60 years of age, respectively. The prevalence of diabetes increased with increasing age (*P* < 0.001). The prevalence of IFG was 8.27%, 10.80% and 11.52% among patients who were 18–40, 40–60, and ≥ 60 years of age, respectively. The prevalence of IFG did not differ significantly according to age (*P* = 0.125).

### Prevalence of hyperglycemia in patients according to ethnicity

The prevalence of diabetes was 9.24% and 14.37% among the Han patients and ethnic minority patients, respectively. The prevalence of diabetes among ethnic minority patients was higher than that among the Han patients (*P* = 0.001). The prevalence of IFG was 9.50% and 9.38% among the Han patients and ethnic minority patients, respectively. The prevalence of IFG did not differ significantly according to ethnicity (*P* = 0.936).

### Risk factors for diabetes among adults with newly diagnosed HIV/AIDS

In a multivariate analysis using a logistic regression model, we analyzed factors associated with the presence of diabetes. Table 2 summarizes the results of the final regression model. Older age, lower CD4 count and minority ethnicity were significantly associated with an increased risk of diabetes. HIV transmission route and sex failed to show an association with the presence of diabetes.

**Table 2:** Identification of risk factors for the presence of diabetes, results of the regression model

**Risk factor**	***P *value**	**Odds ratio**	**95% Confidence interval**
Age,per 20-year increment	<0.001	1.961	(1.587, 2.422)
CD4 count, per decrease of 150 cells/mm^3^	0.017	1.225	(1.036, 1.448)
Minority ethnicity	0.004	1.600	(1.160, 2.207)
Sex	0.165	0.764	(0.523, 1.117)
HIV transmission route			
Sexual contact	0.606	1.000	–
Blood	0.404	0.809	(0.491, 1.332)
Unknown transmission risk	0.326	0.731	(0.391, 1.366)

## Discussion

A national survey of the general adult population in 2008 showed that the prevalence of total diabetes and of pre-diabetes among all Chinese adults was 9.7% and 15.5%, respectively [8]. Our study showed that the prevalence of diabetes and of IFG among Chinese adults with newly diagnosed HIV/AIDS was 10.52% and 9.47% respectively, which suggests that hyperglycemia is also relatively prevalent among newly diagnosed HIV/AIDS patients in China. It also showed that among patients aged 18–40 years the prevalence of diabetes was 7.00% and of IFG 8.27%, indicating that hyperglycemia is even common among the younger newly diagnosed HIV-infected patients. At present, the number of HIV/AIDS patients is still on the rise in China [9]. With the growing number of people living with HIV/AIDS, the number of persons with hyperglycemia and HIV/AIDS is expected to increase. Therefore, newly diagnosed HIV/AIDS patients should be screened for hyperglycemia and this should become an important part of HIV care in China.

Our study showed that there was a high prevalence of diabetes among adults with newly diagnosed HIV/AIDS, and that lower CD4 count was associated with an increased risk of diabetes, since the prevalence of diabetes increased with decreasing CD4 count. This suggests that the presence of diabetes may be related to HIV infection. The underlying cause and origin of diabetes in HIV/AIDS patients may differ from that of the general population [7]. Our study population was newly diagnosed HIV/AIDS patients who had not received ART, hence hyperglycemia among these patients was not caused by antiretroviral drugs. Although the etiology of glucose disorders in HIV persons remains unknown and no study has yet directly confirmed that HIV infection may cause hyperglycemia, current studies have shown that HIV infection is related to cardiovascular diseases and metabolic disorders. Severity of HIV disease has been associated with increased risk of lipo-atrophy, lipo-dystrophy, and cardiovascular disease [10-14]. Together with our study, these associations suggest that HIV-related factors probably contribute to the pathogenesis of IFG and diabetes in HIV-infected patients not using ART. However, our study cannot fully confirm that HIV infection results in hyperglycemia. Therefore, the contribution of HIV-related factors in the development of hyperglycemia still needs further study.

Our study showed that prevalence of diabetes increased with increasing age. Older age was significantly associated with an increased risk of diabetes, increasing the odds by a factor of 1.961. This indicates that older age is a risk factor for diabetes among adults with newly diagnosed HIV/AIDS. Glycemic markers should be monitored regularly in aging HIV-infected patients.

In this study population, the prevalence of IFG was significantly higher among men than among women, and the prevalence of diabetes was slightly higher among men than among women. In addition, the prevalence of diabetes was significantly higher among ethnic minority patients than among the Han patients. Minority ethnicity was significantly associated with an increased risk of diabetes, increasing the odds by a factor of 1.600. However, we did not find a significant association between gender and the presence of diabetes among newly diagnosed HIV/AIDS patients by a logistic regression model; HIV transmission route also failed to show association with the presence of diabetes.

The abnormal glucose metabolism that occurs during the course of ART for HIV has gained much attention in recent years. At present, attention is focused on the relationship between ART and the presence of diabetes. Current studies show a significant relationship between new-onset diabetes and exposure to ART; the incidence of diabetes increased with cumulative exposure to ART. A study showed that the incidence of diabetes in HIV-infected men with ART exposure was greater than four times that of HIV-seronegative men [15]. A Swiss HIV cohort study showed that the incidence of diabetes in patients receiving ART was 4.42 cases per 1000 person-years of follow-up [16]. That study found that current treatment with protease inhibitor- and nucleoside reverse-transcriptase inhibitor-containing regimens was associated with the risk of developing type 2 diabetes [16]. Another study demonstrated that stavudine and zidovudine are significantly associated with diabetes after adjustment for risk factors for diabetes and lipids [6]. A French study showed that the incidence of diabetes in patients receiving ART was 14.1 cases per 1000 person-years of follow-up, and that the occurrence of diabetes was not associated with HIV-related markers [17]. Although antiretroviral drugs have a direct role in the pathogenesis of hyperglycemia in HIV patients, some studies suggest that HIV disease may also be associated with the development of hyperglycemia [5]. One study showed that HIV-infected men with lower nadir CD4 cell counts had an increased risk of incident glucose abnormalities compared with those with higher nadir CD4 cell counts after initiating HIV treatment [15].

Some limitations to our study should be noted. First, potential sample selection bias may have affected the findings. China is a country with a large territory and an extremely large population. The HIV epidemic is serious in some areas and among some most-at-risk populations. There may exist differences in prevalence of hyperglycemia among patients with different demographic characteristics and lifestyle. Second, some studies have demonstrated body-mass index, lifestyle, and family history of hyperglycemia are associated with the prevalence of IFG and diabetes [6,8,15], but these factors were not assessed in our study. Therefore, we were not able to determine the association between these factors and the prevalence of hyperglycemia. Furthermore, if these variables had been controlled for, some variables such as CD4 count may not have remained significant in the logistic regression model. Third, impaired glucose tolerance (IGT) and early insulin resistance are particularly prevalent in HIV patients (10%-25%), especially in those receiving ART containing protease inhibitors [18]. However, we did not do an oral glucose-tolerance test for the patients, thus we can not estimate the prevalence of IGT in HIV patients. In this study, we used only one fasting glucose level to diagnose diabetes, this may have led to over-reporting of the true prevalence of diabetes. In addition, the design of the study was observational; we were able to examine potential associations but were unable to assess causation, so it is not clear if the diabetes preceded the HIV infection or vice versa.

## Conclusions

Hyperglycemia is highly prevalent among adults with newly diagnosed HIV/AIDS in China. Older age, lower CD4 count and minority ethnicity are associated with increased risk of diabetes. It is necessary to routinely screen IFG and diabetes among newly diagnosed HIV/AIDS patients. We strongly recommend that all newly diagnosed HIV/AIDS patients should be routinely evaluated for hyperglycemia both before and after initiating HIV treatment. Further research is needed to explore the impact of HIV infection on the development of hyperglycemia in HIV-infected patients.

## Competing interests

The authors declare that they have no competing interests.

## Authors’ contributions

YZS and HZL conceived of the study, and participated in its design and coordination. YZS performed the statistical analysis and drafted the manuscript. ZYW, LL, RFZ and YFZ participated in data collection. All authors read and approved the final manuscript.

## Pre-publication history

The pre-publication history for this paper can be accessed here:

http://www.biomedcentral.com/1471-2334/13/79/prepub
